# Lipid accumulation and oxidation in glioblastoma multiforme

**DOI:** 10.1038/s41598-019-55985-z

**Published:** 2019-12-20

**Authors:** Bouchra Taïb, Amine M. Aboussalah, Mohammed Moniruzzaman, Suming Chen, Norman J. Haughey, Sangwon F. Kim, Rexford S. Ahima

**Affiliations:** 1 Department of Medicine, Division of Endocrinology, Diabetes and Metabolism, Johns Hopkins University, Baltimore, Maryland USA; 2 Department of Mechanical & Industrial Engineering, University of Toronto, Toronto, Canada; 3 Department of Neurology, Johns Hopkins University, Baltimore, Maryland USA; 4 Department of Neuroscience, Johns Hopkins University, Baltimore, Maryland USA

**Keywords:** Cancer in the nervous system, Cancer metabolism

## Abstract

Glioblastoma multiforme (GBM) is the most common and lethal primary malignant brain tumor in adults. Despite the multimodal standard treatments for GBM, the median survival is still about one year. Analysis of brain tissues from GBM patients shows that lipid droplets are highly enriched in tumor tissues while undetectable in normal brain tissues, yet the identity and functions of lipid species in GBM are not well understood. The aims of the present work are to determine how GBM utilizes fatty acids, and assess their roles in GBM proliferation. Treatment of U138 GBM cells with a monounsaturated fatty acid, oleic acid, induces accumulation of perilipin 2-coated lipid droplets containing triglycerides enriched in C18:1 fatty acid, and increases fatty acid oxidation. Interestingly, oleic acid also increases glucose utilization and proliferation of GBM cells. In contrast, pharmacologic inhibition of monoacylglycerol lipase attenuates GBM proliferation. Our findings demonstrate that monounsaturated fatty acids promote GBM proliferation via triglyceride metabolism, suggesting a novel lipid droplet-mediated pathway which may be targeted for GBM treatment.

## Introduction

Glioblastoma multiform (GBM), also known as grade 4 astrocytoma, is the most common and deadly primary tumor type in the adult human brain^1^. The standard treatments for patients with GBM are surgical resection, chemotherapy and radiation^2^. Unfortunately, most patients eventually develop a recurrence of GBM, and the median overall survival is 10–14 months^3^. It is well established that cancer cells adopt alternative metabolic pathways, and this metabolic reprogramming can lead to treatment resistance^4^. Understanding the metabolic pathways involved in regulating GBM proliferation may lead to better treatment toward improving the overall survival of GBM patients.

To generate the energy needed for cellular processes, healthy cells rely mainly on mitochondrial oxidative phosphorylation, typically maintaining a relatively low glycolytic rate. Conversely, cancer cells depend predominantly on energy produced through glycolysis and the resulting lactic acid fermentation. This phenomenon, known as “the Warburg effect”, was long considered a fundamental metabolic difference between cancer cells and normal cells. Lipid metabolism is also altered in rapidly proliferating cells^5,6^, and increased lipogenesis has been considered as another metabolic hallmark of cancer cells. Consistent with this, glioma tissues have been shown to have enhanced lipid synthesis^7^, and NMR studies have revealed a correlation between lipid resonance spectra of GBM extracts and the grade of malignancy^8^. In addition to increasing *de novo* lipid synthesis, cancer cells may also use exogenous fatty acids to fuel their growth^9^. Oral administration of stable isotope labeled [^13^C] fatty acids to mice by gastric gavage showed a strong [^13^C] enrichment in gliomas and only low level of [^13^C] enrichment in the capillary endothelial cells of normal brain^10^. These results were supported by previous study showing that the blood-brain barrier in experimental brain metastases was impaired^11^. Moreover, lipids, including fatty acids (FAs), triglycerides (TAGs), and phospholipids (PLs), have emerged as mediators of signal transduction involved in many physiological responses^12^. However, the mechanism by which lipids participate in the progression of brain tumors is still elusive.

Within the cell, FAs can be esterified with glycerol leading to the formation of TAGs which are then stored in lipid droplets (LDs), a dynamic and structured cellular organelle. The TAG core of a LD is enclosed by a PL layer that is coated with lipid-binding proteins^13,14^, of which perilipin (PLIN) family proteins are among the most abundant. In mammals, there are five major PLIN proteins (PLIN 1 through 5). PLIN 2 (also called adipose differentiation-related protein - ADRP) and PLIN 3 are ubiquitously expressed, while PLIN 1, 4, and 5, have limited tissue expression. Of the five known PLIN proteins, PLIN 2 is exclusively associated with lipid droplets^15^.

LDs are found in tissues with high lipid turnover, such as adipose and liver tissues^16,17^, and are often associated with the development of metabolic diseases linked to lipid accumulation, including obesity and diabetes. LDs are commonly viewed as energy storage organelles since the TAGs stored in LDs can readily be hydrolyzed into free FAs and glycerol for energy production by patatin-like phospholipase domain-containing protein 2 (PNPLA2), a member of the PNPLA family, also known as adipose triglyceride lipase (ATGL)^18^ and monoacylglycerol lipase (MAGL)^14^. In the central nervous system, LD levels are barely detectable, however, recent studies show that neurons and glia accumulate LDs under disease conditions such as brain tumors^19,20^.

Importantly, MAGL is upregulated in many aggressive human cancer cells and primary tumors, and acts as a critical regulator of tumorigenesis, migration, invasion, and metastasis by providing lipolytic sources of free FAs for synthesis of oncogenic signaling lipids^21^. Monounsaturated oleic acid and saturated palmitic acid are the most abundant long chain fatty acids (LCFAs) found in plasma. However, many studies show that only unsaturated fatty acids level are high in human intracranial tumors, including glioma^7^. Thus, in this study, to further delineate how GBM cells metabolize exogenous LCFAs, we focused on investigating effects of monounsaturated LCFA, oleic acid, on GBM metabolism and proliferation.

## Results

### Oleic acid increases accumulation of TAG enriched lipid droplets in human GBM cells

**Figure 1:**
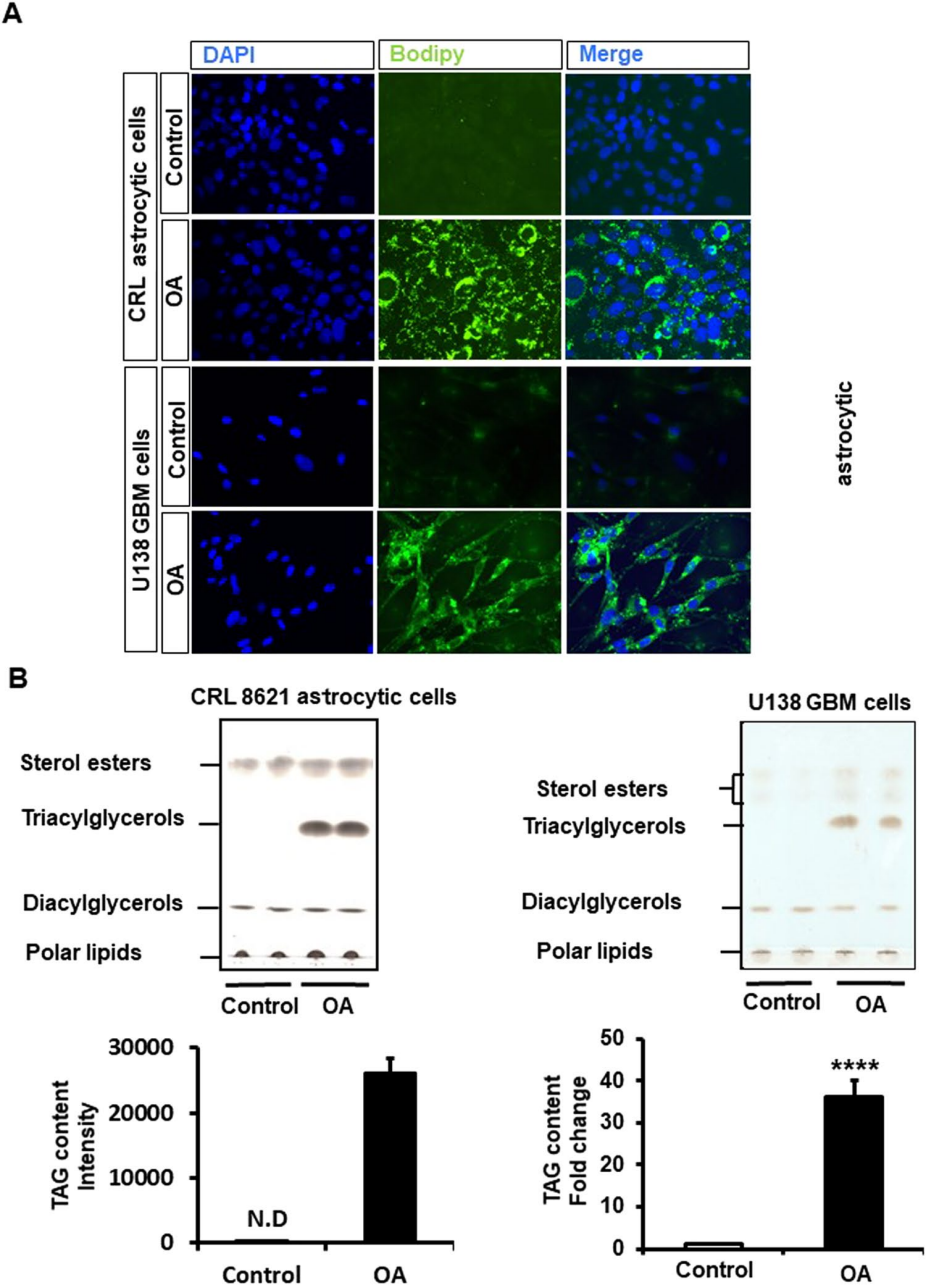
Effect of oleic acid treatment on LDs and TAGs accumulation in U138 human GBM cells and CRL 8621 human astrocytes. (**A**) LD accumulation in response to oleic acid (OA, 100 μM) was revealed using Bodipy staining, DAPI (blue) and fluorescence (green). (**B**) Lipid levels were determined by thin layer chromatography (TLC) and quantified using Image J software. Relative levels of fatty acids were normalized by protein content. Values were means ± SE, N = 3 independent experiments for TLC. N.D: not detected. Statistical analyses were performed with unpaired Student’s t-test. ****p < 0.0001 versus control.

We used the established human malignant GBM cell line U138-MG to examine the effect of oleic acid on LDs accumulation. Cells were treated with oleic acid (100 μM) for 24 hours (hrs) in the presence of 5 mM glucose, and normal astrocytes cell line CRL 8621 was used as a control. Bodipy staining showed accumulation of LDs in U138 GBM cells as well as in normal astrocytic cells in response to oleic acid treatment (Fig. 1A). The analysis of lipid content by thin-layer chromatography (TLC) showed that oleic acid treatment increased fatty acid incorporation into TAGs in both GBM cells and normal astrocytic cells compared to the control condition (Fig. 1B).

### Oleic acid increases proliferation of GBM cells but not normal astrocytes

**Figure 2:**
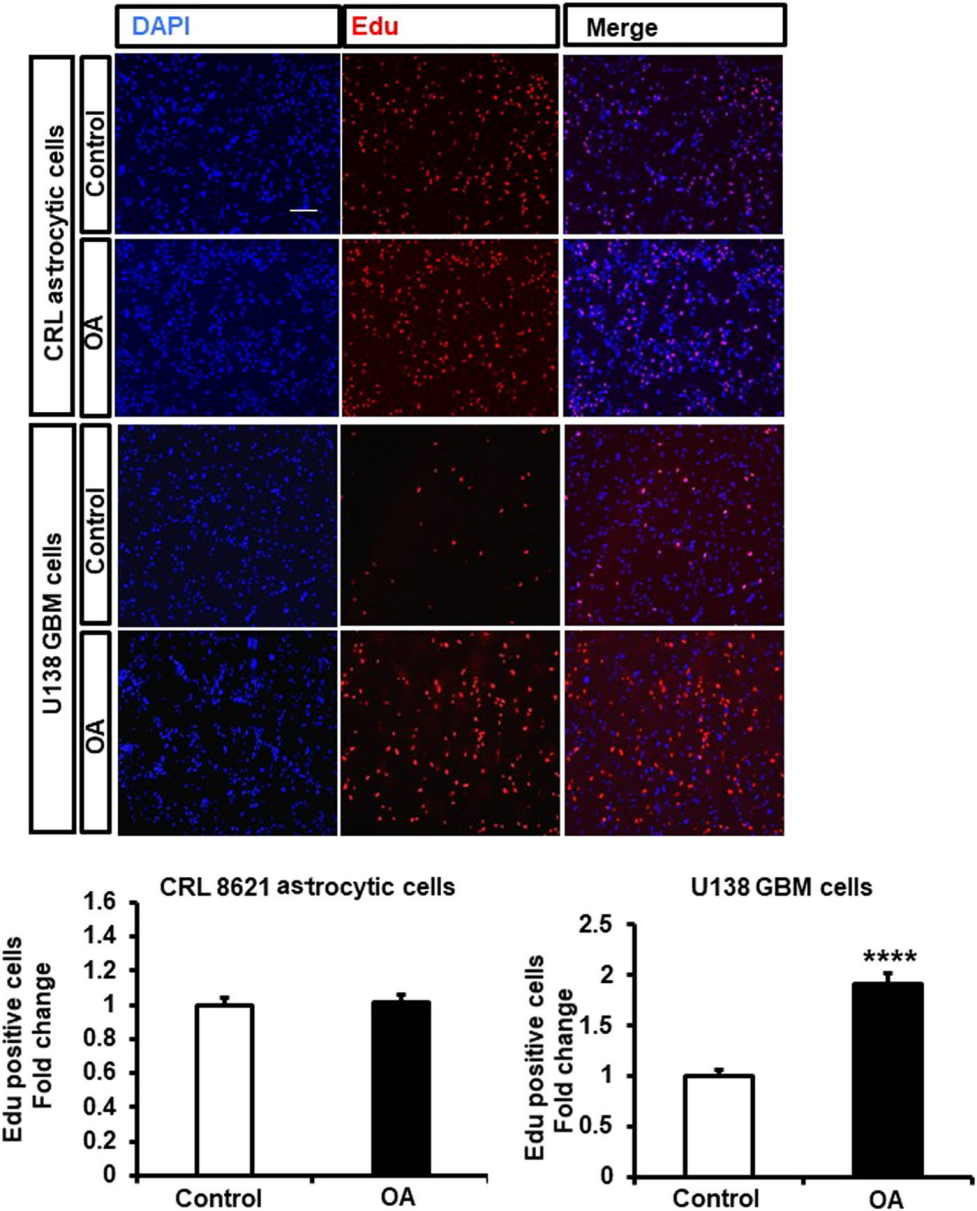
Oleic acid stimulates U138-MG GBM cells but not CRL 8621 astrocytes proliferation. (**A**) EdU proliferation test was performed in the presence of oleic acid (OA, 100 μM). Proliferating cells were detected via EdU (red). DAPI stained nuclei in blue. Merged view of EdU (red) and DAPI (blue). The images are representative of the data obtained. Quantification was performed using Image J software. Values were means ± SE. N = 3 independent experiments. Statistical analyses were performed with unpaired student’s t-test. ****p < 0.0001 versus control. (Scale bar = 0.1 mm).

Cell proliferation is a highly energetic process tightly associated with cellular energy metabolism. We therefore investigated whether oleic acid influenced the proliferation of GBM cells and normal astrocytes. Using the EdU assay, we assessed proliferation after 24 hrs treatment with oleic acid and found that oleic acid enhanced cell proliferation of U138 but not normal astrocytes CRL 8621 (Fig. 2). In contrast, we found that oleic acid did not affect GBM cell migration (Supplementary Fig. S2).

### Effect of oleic acid supplementation on lipid composition in human GBM cells

**Figure 3:**
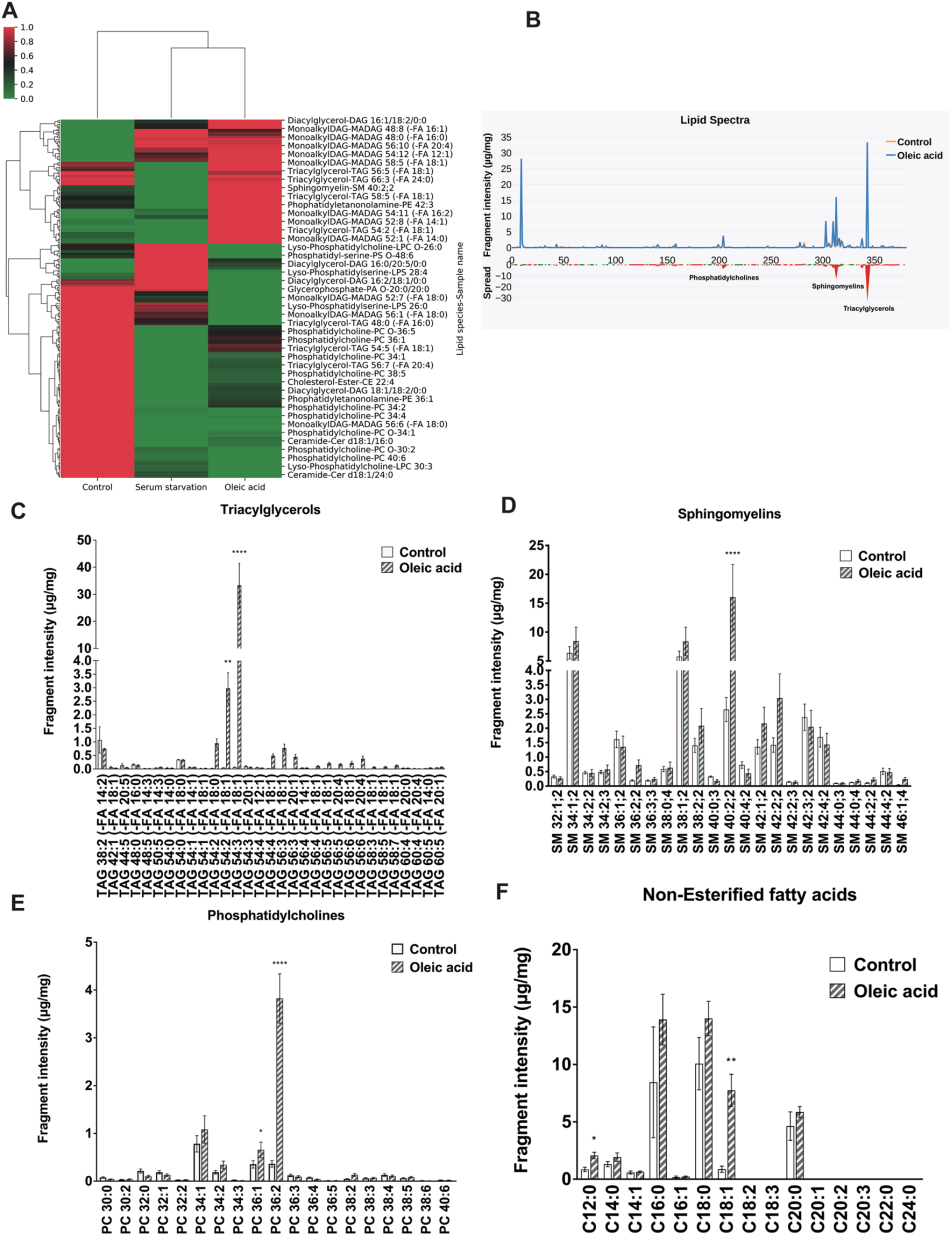
Oleic acid modifies lipid composition in U138 human GBM cells. (**A**) Hierarchical clustering heatmap analysis of lipids in U138-MG glioma cells in response to serum starvation and oleic acid treatment. The columns represent sample’s conditions and the rows indicate lipids. Each colored cell on the map corresponds to a concentration value. The distances among the clusters are presented by dendrograms between the clusters and indicate similarity between lipids species. (**B**) Reconstructed Lipid species spectra in response to oleic acid treatment. Intensity of lipids species is presented as peaks in (µg/mg). The differences between control and oleic acid treatment are presented as a red spread. (**C**) Triacylglycerols. (**D**) Sphingomyelins. (**E**) Phosphatidylcholine. (**F**) Non-esterified Fatty acid composition in U138 GBM cells in response to 24 hrs oleic acid treatment versus control. Relative levels of fatty acids were normalized by protein content. Values were means ± SE. N = 3 for mass spectrometry. Results are presented in (µg/mg). Statistical analyses were performed with unpaired Student’s t-test. *, **, and ****p < 0.05, 0.01, 0.0001 versus control.

In order to quantitatively evaluate the effect of oleic acid supplementation on lipid composition in GBM cells, we performed mass spectrometry comparing control cells with 24 hrs serum starved cells, and 24 hrs serum starved cells supplemented with oleic acid (100 µM). A total of 382 fatty acid species were identified from five major lipid classes including ceramides, PLs, DAGs, TAGs, and sphingomyelins (SMs) (data not shown). Distinct patterns for 3 separate clusters of lipids were identified in each treatment condition (Fig. 3A). Under the control condition, the most abundant lipid classes present were PLs, DAGs and TAGs. Serum starvation induced a switch in the pattern of lipids, characterized by an increase in phosphatidylserines and DAGs and a decrease in sphingolipids. Finally, as expected, oleic acid treatment resulted in an increase in TAGs (Fig. 3A) (Supplementary Fig. S3), SMs and phosphatidylcholines (PCs) (Fig. 3B). A more detailed analysis of the fatty acid composition showed significant increases in TAGs 54:2 and 54:3, (Fig. 3C), SM 40:2 (Fig. 3D), PC 36:1 and PC 36:2 (Fig. 3E) in cells treated with oleic acid. Other less abundant TAG and PC species did not show changes in response to oleic acid treatment (Supplementary Table 1). In addition, analysis of the non-esterified fatty acids composition showed that under control conditions, GBM cells were enriched in palmitic acid (C16:0), stearic acid (C18:0) and oleic acid (C18:1), while oleic acid treatment selectively increased C18:1 level (Fig. 3F).

### Oleic acid regulates lipogenic genes in human GBM cells

**Figure 4:**
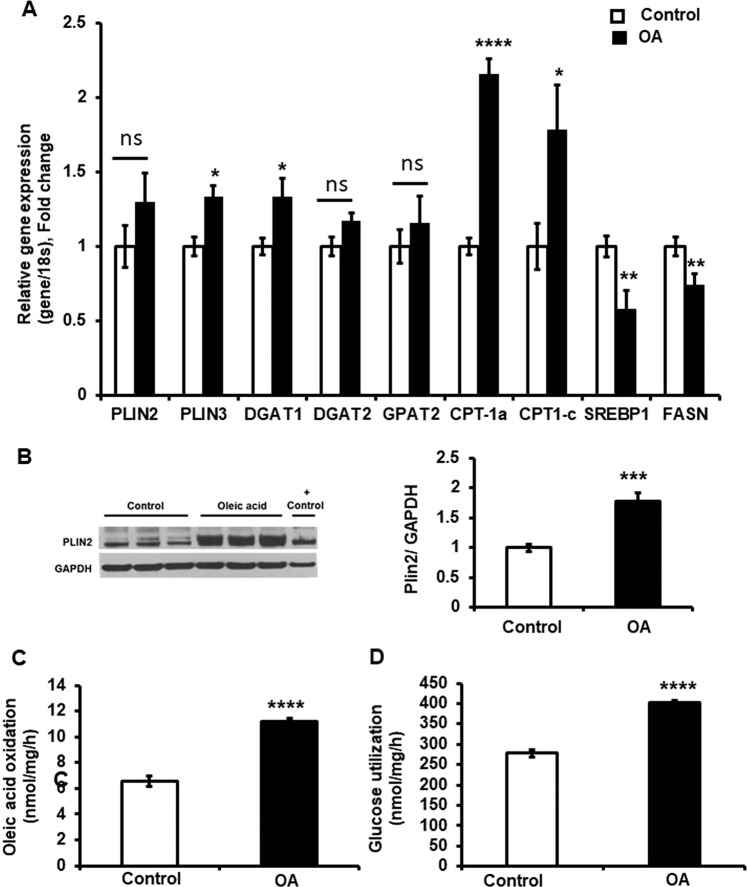
Oleic acid increases β oxidation, glucose utilization and expression of genes involved in LCFA metabolism in U138 GBM cells. (**A**) Gene expression was determined by qPCR and mRNA levels were normalized to 18 S levels. Perilipin (PLIN), Diacylglycerol O-acyl-transferase (DGAT), Carnitine acyltransferase1 (CPT-1), Sterol regulatory element-binding proteins (SREBP), Fatty acid synthase (FASN). (**B**) Cropped western blot images and quantitation of PLIN2 and GAPDH protein levels from cell lysates. Human hepatoma (VL17A) cell line was used as a positive control (+control). Full length blots are in Supplementary Fig. 1. N = 3 independent experiments in triplicate. (**C**) Oleic acid oxidation, and (**D**) glucose utilization were measured using a ^14^C-oleic acid and ^3^H-glucose). N = 3 independent experiments in triplicate. Values were means ± SE. Statistical analyses were performed with unpaired Student t-test *, **, ***, and ****p < 0.05, 0.01, 0.001, and 0.0001 versus control. OA: Oleic acid.

Next, we determined whether the oleic acid-mediated increased TAGs correlated with changes in the expression of genes involved in lipid metabolism. Oleic acid treatment of U138-MG GBM cells increased expression of diacylglycerol acyltransferase isoform 1 (DGAT1), an enzyme involved in the terminal step of TAGs synthesis, but not DGAT2. Oleic acid also increased the expression of carnitine palmitoyl transferase-1 (CPT-1) isoforms a and c, the main isoforms of the enzyme responsible for fatty acid transport into the mitochondria for β oxidation, while the expression of glycerol-3-phosphate acyltransferase 2 (GPAT2), the enzyme involved in de novo synthesis of TAGs was not affected. PLIN3 mRNA expression was increased, while PLIN2 was unaffected. In contrast, the expression level of fatty acid synthase (FAS) and sterol regulatory element-binding protein-1c (SREBP1c), which are involved in de novo lipogenesis, were decreased in response to oleic acid treatment (Fig. 4A). The western blot analysis showed that oleic acid treatment increased PLIN2 protein expression in U138 GBM cells (Fig. 4B).

### Oleic acid increases fatty acid oxidation and glucose utilization in human GBM cells

Under serum-free culture conditions fatty acid oxidation is the primary catabolic pathway in human GBM cells^22^. To determine whether oleic acid treatment changes the energy metabolism of U138-MG GBM cells, we measured fatty acid β-oxidation using [^14^C]oleic acid and glucose utilization using [^3^H] glucose. The results showed significant increases of both β-oxidation and glucose utilization (Fig. 4C,D) in response to oleic acid treatment, indicating an increase in catabolic pathways.

### GBM cell proliferation induced by oleic acid requires TAGs hydrolysis

**Figure 5:**
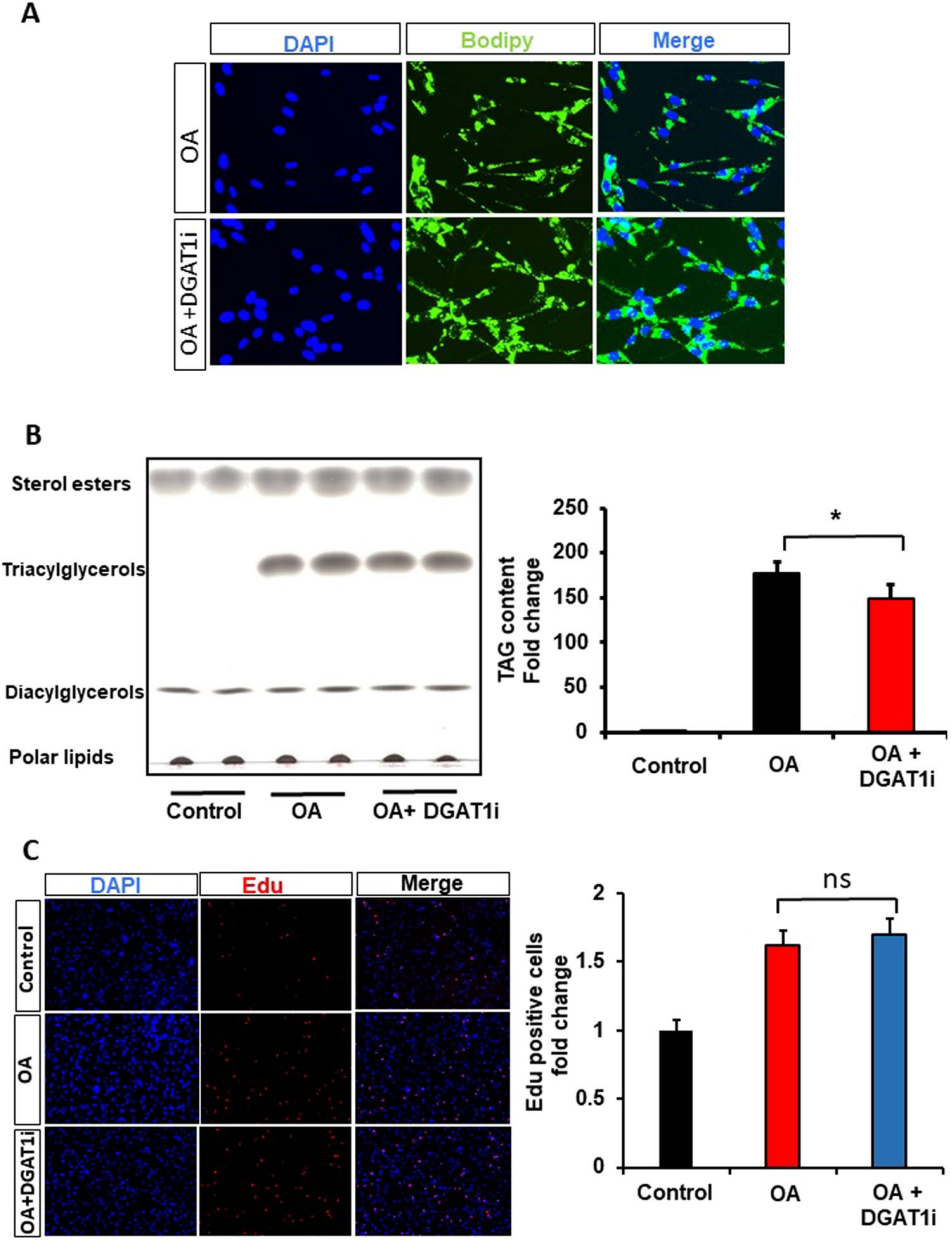
DGAT1 inhibitor decreases slightly TAGs accumulation without abolishing the effect of oleic acid on U138 GBM cell proliferation. (**A**) LD accumulation in response to DGAT1 inhibitor (DGAT1i,  PF-04620110, 3 µM) was revealed using Bodipy staining, DAPI (blue) and fluorescence (green). (**B**) Lipid levels were determined by thin layer chromatography (TLC) and quantified using Image J software. (C) EDU proliferation test was performed in presence of oleic acid, (OA, 100 µM), in response to (PF-04620110, 3 µM). Proliferating cells were detected via EDU (red). DAPI stained nuclei in blue. Merged view of EdU (red) and DAPI (blue). N = 3 independent experiments. Statistical analyses were performed with unpaired Student’s t-test *p < 0.05 versus control.

Studies have shown that TAGs storage is important for growth of cancer cell^23^. To further elucidate the mechanism by which oleic acid induces GBM proliferation (Fig. 2), we investigated whether TAGs were involved. We first examined whether the inhibition of DGAT1 enzymes would lead to a decrease of TAGs synthesis. We found that inhibition of DGAT1 by PF04620110 (3μM ) led to an accumulation of LDs by Bodipy staining (Fig. 5A), and TAGs accumulation was decreased in U138 GBM cells (Fig. 5B). However, this decrease of TAGs synthesis did not affect GBM proliferation (Fig. 5C). We also treated GBM cells with etomoxir (200 µM), a potent CPT1 pharmacological inhibitor, in the presence of oleic acid and confirmed a significant inhibition of cell proliferation (Supplementary Fig. S4).

**Figure 6:**
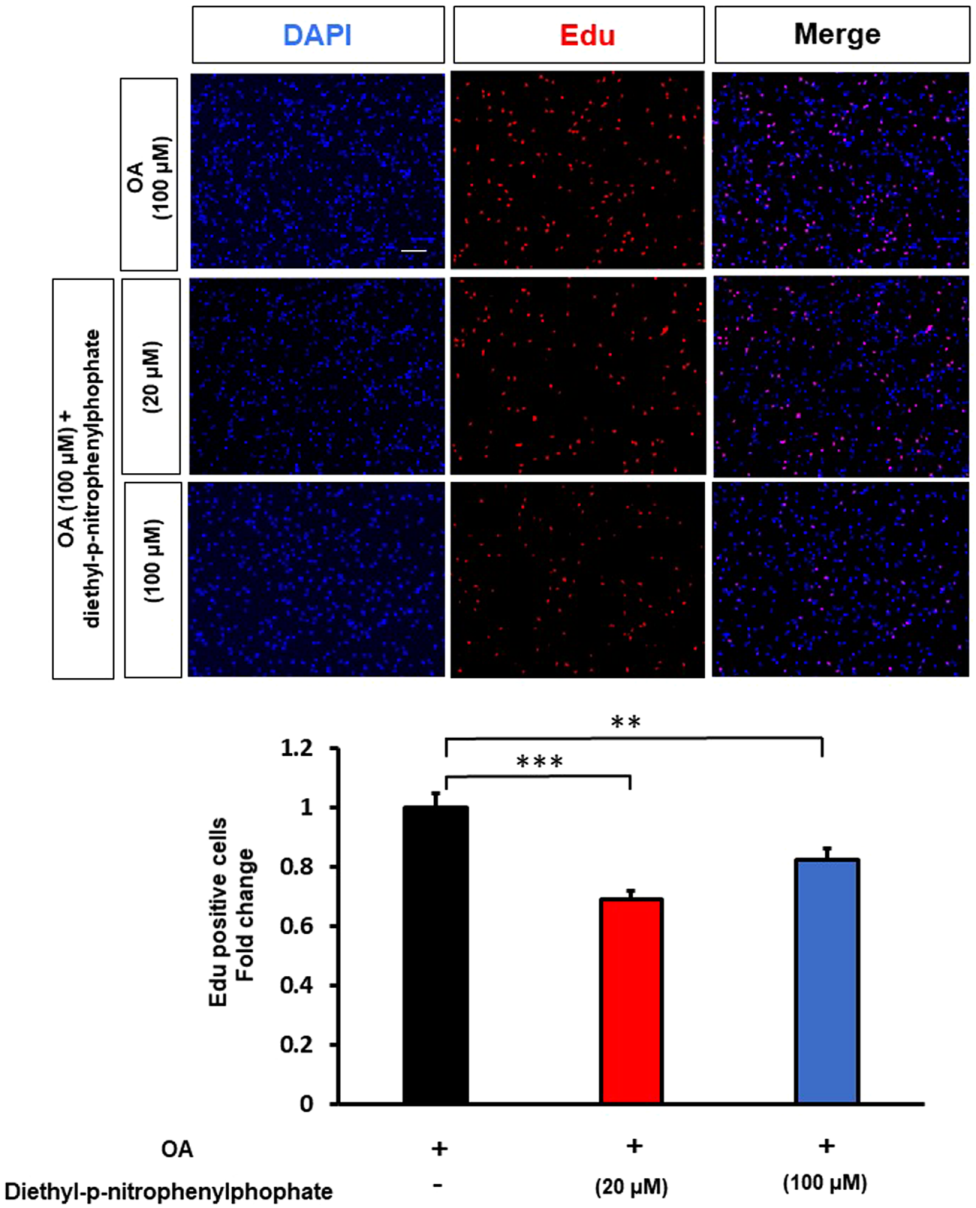
General lipases inhibition attenuates the effect of oleic acid on GBM cell proliferation. EDU proliferation test was performed in presence of oleic acid (OA, 100 µM), in response to different concentrations of the general lipase inhibitor diethyl-p-nitrophenylphosphate (20 µM, 100 µM). Proliferating cells were detected via EDU (red). DAPI stained nuclei in blue. Merged view of EdU (red) and DAPI (blue). N = 3 independent experiments. Statistical analyses were performed with unpaired Student’s t-test **, and ***p < 0.01 and 0.001 versus control. (Scale bar = 0.1 mm).

**Figure 7:**
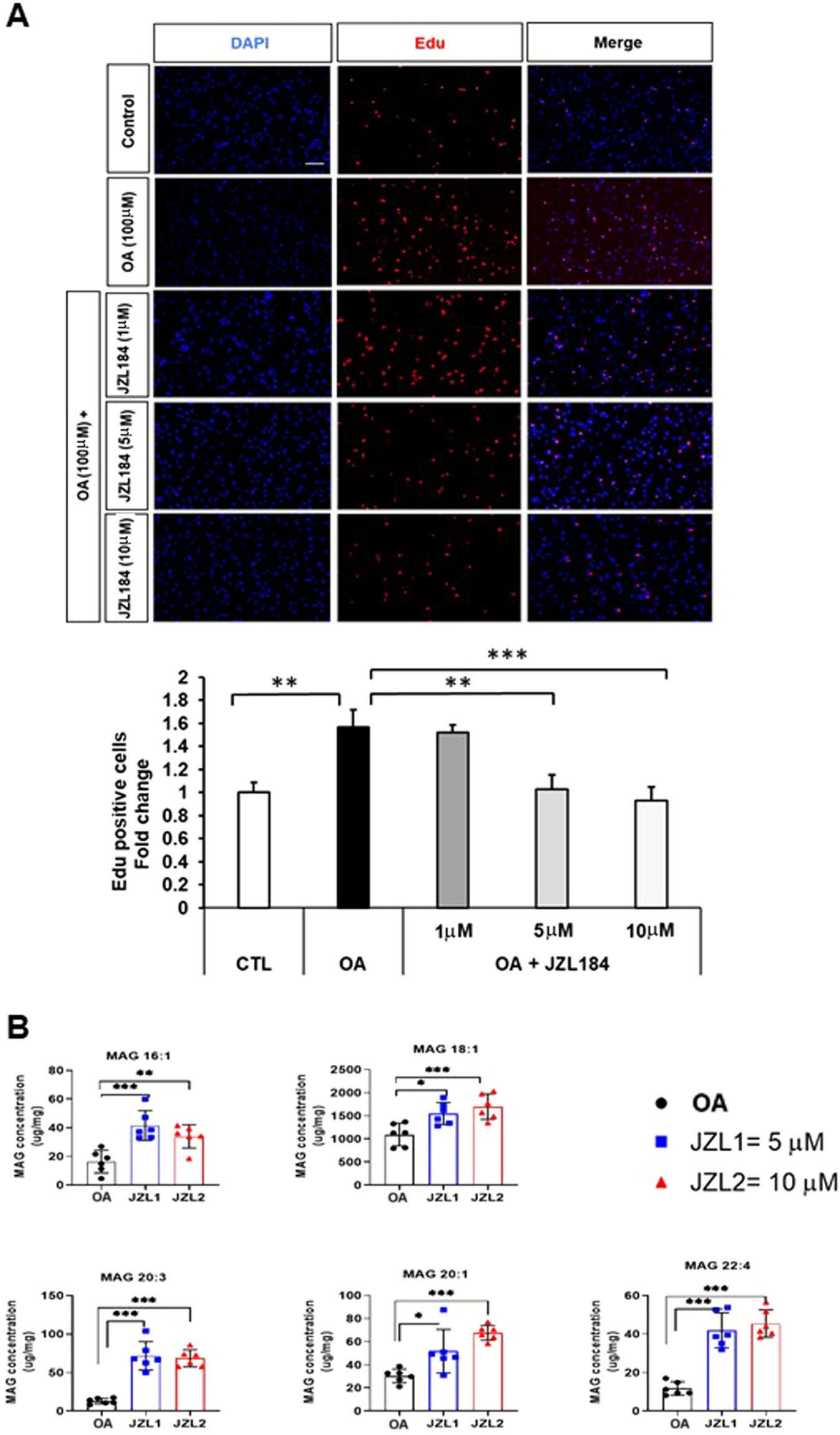
MAGL inhibition attenuates the effect of oleic acid on GBM cell proliferation. (**A**) EDU proliferation test was performed in presence of oleic acid (OA, 100μM ), in response to different concentrations of JZL184 (1, 5, and 10 µM). Proliferating cells were detected via EDU (red). DAPI stained nuclei in blue. Merged view of EdU (red) and DAPI (blue). N = 3 independent experiments. Statistical analyses were performed with unpaired Student’s t-test **, and *** p < 0.01 and 0.001 versus control. (Scale bar = 0.1 mm). (**B**) U138 GBM cells show elevations in MAG species in presence of JZL184 (5 and 10 µM) N = 3 for mass spectrometry. Results are presented in (µg/mg). Statistical analyses were performed with unpaired Student’s t-test. *, **, and ***p < 0.05, 0.01, 0.001 versus control.

The hydrolysis and mobilization of FAs from TAGs is controlled by specific lipases. Using the general lipase inhibitor, diethyl-p-nitrophenylphosphate, at concentrations of 20 µM and 100 µM, we observed a significant inhibition of GBM U138 cell proliferation (Fig. 6). Moreover, addition of glycerol (0.6 M) did not abolish the effect of diethyl-p-nitrophenylphosphate (Supplementary Fig. S5A). Previous studies have shown that MAGL is highly expressed in aggressive cancer cells and controls the fatty acid release from lipid stores^21^. Thus, blockade of MAGL by a selective inhibitor JZL184 (5 µM, 10 µM, 24 hrs) reduced GBM cell proliferation as shown by EdU assay (Fig. 7A). Addition of glycerol did not attenuate the effect of JZL184. (Supplementary Fig. S5B). Moreover, the mass spectrometry quantitative analysis data confirmed that JZL184 treatment produced significant elevations in the levels of several MAGs including MAGs 16:1, 18:1, 20:1, 20:3, and 22:4 (Fig. 7B). Together, our results demonstrate that TAGs are involved in oleic acid-induced proliferation in GBM cells, and MAGL plays an important role in GBM proliferation.

## Discussion

Despite aggressive therapy, the treatment of GBM is difficult and the overall mortality rates remain high. Further research is necessary to better understand the factors involved in the pathogenesis of GBM. LDs are energy storage organelles abundant in hepatocytes and adipocytes. In the brain, using electron microscopy and fluorescence imaging, it has been observed that tumor tissues from GBM patients contain large amounts of LDs^24^. We found that 24 hrs treatment of U138 GBM cells with oleic acid was able to induce accumulation of LDs enriched in TAGs, the same was true for CRL 8621 astrocytic cells. It has been known that saturated FFAs are pro-apoptotic, whereas unsaturated FFAs stimulate cell proliferation in some cancer types such as breast cancer^25^. In this study, we showed that oleic acid induces proliferation in human GBM cells, but not in normal astrocytic cell line CRL 8621. PLIN2 is a protein that is associated with LDs, and immunoblot analysis further showed treatment of GBM cells with oleic acid also increased PLIN2 protein. Moreover, It has been reported that activation of PLIN2 in GBM cells is driven by hypoxia^26^. In our study, we showed that PLIN2 protein activation in GBM cells occurs under normoxic conditions in the presence of excess exogenous fatty acid. We further confirmed that the lipid profile of U138 GBM cells changes in response to the nutrient composition. Lipidomic analysis showed that the human GBM cell line U138, predominantly accumulates five main classes of lipids: Ceramides, PLs, DAGs, TAGs, and SMs. Lipids largely serve as structural components of cellular membranes in addition to other functions^27^. The presence of different PL species is consistent with previous studies showing higher amounts of PLs in the serum of patients with various primary brain tumors^28^. Likewise, it has been reported that under lipid-restricted growth conditions, cancer cells exhibit increased reliance on *de novo* FA synthesis in order to maintain their growth and survival^29^. In our study, we identified multiple changes in lipidomic profiles in cells in response to serum and lipids deprived conditions especially in the profiles of PLs, DAGs and TAGs. Hence, we speculate that these changes in lipid species may indicate putative metabolic pathways mediating the growth of GBM cells. Mass spectrometry analysis, after oleic acid treatment, shows that the increased Non-esterified FAs and TAGs were enriched in C18:1 suggesting that these changes were derived from the elevated concentration of exogenous oleic acid, rather than *de novo* lipogenesis. The expression level of DGAT1, which is predominantly involved in TAG synthesis from exogenous FAs^30^, was significantly increased, whereas genes involved in *de novo* lipogenesis, such as DGAT2 and GPAT2, were not affected by oleic acid treatment. The composition of PLs showed that PC species PC36:1 and PC36:2 increased in response to oleic acid treatment. Despite the increase of PCs synthesis is frequently observed in cancer cells in response to fatty acid substrates, the effects of oncogenic transformation on PCs synthesis are inconsistent. However, previous data suggest that inhibition of FAs synthesis could inhibit tumor growth by suppressing the synthesis of PCs and other PLs^31^. Finally, among the different SM species present in GBM cells, only SM 40:2 was increased in response to oleic acid treatment. The role of different molecular species of SMs in normal brain cells is not understood, but in transformed tissues, changes in SMs vary depending on the type of cancer^32^.

LCFAs are normally balanced between esterification and oxidation. In GBM cells, it has been shown that fatty acid oxidation contributes to energy production^5^, our finding show that GBM cells increase their level of fatty acid oxidation in response to oleic acid treatment. This result is supported by the increase of gene expression level of the most common isoforms of CPT-1 found in the brain, CPT-1a and CPT-1c. Recent studies showed that CPT-1c is upregulated in human gliomas^33^, and that high-grade glioblastoma is associated with aberrant expression of CPT-1a and CPT-1c^34^. However, we and others have previously demonstrated that CPT-1c is not involved in LCFAs oxidation^35^, and the role of CPT-1cin fatty acid metabolism still not fully understood unlike CPT-1a^36,37^. These results highlight an important aspect of metabolic reprogramming of fatty acid metabolism in GBM cells.

Interestingly, we found that U138 GBM cells increase glucose utilization in response to oleic acid treatment. FFAs have been shown to inhibit glucose uptake in non-tumor tissues^38^, however, our data are consistent with previous *in vitro* studies showing that this regulatory mechanism was not operative in tumor cells^39^. In fact, it has been shown that in Ehrlich ascites tumor cells, when exogenous FFA palmitic acid was added in the media, the total incorporation of glucose radioactivity into cellular lipid esters was doubled^39^. To the best of our knowledge, our study shows for the first time that GBM cells simultaneously increase their glucose utilization and fatty acid oxidation in the presence of exogenous FFA. It is tempting to speculate that exogenous fatty acids enhance glucose utilization leading to an increased production in lactate which acidifies the tumor environment provoking a local inflammatory response needed for tumor progression^40^. However, additional studies are necessary to confirm this hypothesis and to understand how exogenous FFAs influence glucose utilization in GBM cells.

The use of etomoxir inhibits GBM proliferation suggesting that the increased oleic acid oxidation in response to oleic acid treatment is involved in proliferation. Another study showed that inhibition of fatty acid oxidation decreased proliferation in primary-cultured cells isolated from human glioma and prolonged survival in a syngeneic mouse model of malignant glioma^22^. In our study, etomoxir decreased proliferation of normal astrocytic cells CRL 8621. Despite their benign nature, these immortalized cells proliferate in culture under the right conditions,  our results showed that the astrocytic cell line CRL 8621 also requires fatty acid oxidation to maintain their basal proliferation rate.

To further investigate whether the effect of oleic acid on GBM proliferation involves TAGs, we inhibited the hydrolysis of TAGs to FFAs. Knockdown of ATGL did not show a significant effect on proliferation (data not shown). Contradicting evidence between *in vitro* and *in vivo* studies have been reported regarding the ambiguous role of ATGL in cancer progression and proliferation. *In vitro* studies showed that ATGL upregulation contributes to the aggressiveness and proliferation of high-grade tumors^41^, while the loss of ATGL was detected in several forms of human cancer^42^. Treatment with diethyl-p-nitrophenylphosphate, a general lipase inhibitor, resulted in a significant decrease in GBM proliferation. While PNPLA2/ATGL serves as the main TAG hydrolase in most peripheral tissues, a recent study found that DDHD2 is the principal triglyceride hydrolase in the central nervous system. Genetic deletion of this enzyme in mice leads to ectopic LD accumulation in neurons throughout the brain^43^. This result may explain why in our study, ATGL knockdown did not affect GBM proliferation.

Furthermore, GPIHBP1, a GPI-anchored protein of capillary endothelial cells which binds LPL to form a crucial complex for the lipolytic process, is assumed to be highly expressed in heart and brown adipose tissue^44^, but absent from the capillaries of the brain^45^. However, a recent study showed that GPIHBP1 is expressed in the capillaries of mouse and human gliomas, and seems to be involved in LPL production. NanoSIMS imaging revealed that GPIHBP1 expression in gliomas facilitates TRL processing and provides a source of lipid nutrients for glioma cells^10^. Collectively, these data suggest that TAGs hydrolysis is involved in GBM proliferation. However, additional studies are necessary to determine which specific lipases mediate TAGs hydrolysis in GBM cells.

In summary, our results demonstrate that in response to exogenous monounsaturated LCFA oleic acid, GBM cells adapt their lipid composition by increasing TAGs accumulation, FA esterification and oxidation. Oleic acid also stimulates GBM proliferation through a mechanism that involves MAGL. Given the critical role of lipids in cell membrane formation and signaling transduction, the identification of the key players involved in lipid metabolism reprogramming could open a new window for novel therapeutic strategies for GBM.

## Experimental Procedures

### Reagents

Culture media Eagle’s Modified Essential Medium (EMEM) was purchased from American Type Culture (ATCC), USA, and fetal bovine serum (FBS) was from Thermo-fisher. Radioactive tracers were from PerkinElmer Life Science, and all other reagents were from Sigma, unless otherwise noted.

### Cell culture and treatment

U-138 MG (ATCC® HTB-16™) and CRL-8621 cell lines (ATCC, USA) were grown in (EMEM) 5 mM glucose supplemented with 10% FBS, and 1% penicillin/ streptomycin at 37 °C in 5% CO_2_. Cells were used at a  density of 60% confluence for metabolic experiment and 80% for lipid extraction.

To assess the effect of oleic acid in GBM, U138-MG glioma cells were seeded in culture media and grown for 24 hrs, then maintained in EMEM (5 mM glucose) without serum in the presence or absence of oleic acid (0.1 mM) precomplexed to BSA 0.13% at the ratio (5:1) for 24 hrs. Different doses of diethyl-p-nitrophenylphosphate (20 µM, 100 µM), JZL184 (1 µM, 5 µM, 10 µM) (selleckchem), and Etomoxir (200 µM) (selleckchem) were used to inhibit respectively TAG hydrolysis, MAG hydrolysis and CPT1. JZL184 was dissolved in dimethyl sulfoxide (DMSO), diethyl-p-nitrophenylphosphate was dissolved in ethanol, and Etomoxir was dissolved in water.

### Bodipy staining

U138 cells were plated in Millicell EZ chamber (Millipore) at a density of 2 × 10^4^ cells/well in 0.5 mL/well medium (for 8-chambered slides) for 24 hrs, then incubated in serum deprived media (5 mM glucose) during 24 hrs in presence of 0.1 mM oleic acid pre-complexed to 0.13% BSA. CRL 8621 were plated on coverslip in 12 well plate at the density of 1.4 × 10^5^cells. Thirty min before the end of the incubation, Bodipy (life sciences cat#: D-3922) was added in the media at the concentration of (1 μM) and cells were then fixed and stained for DAPI (blue) to reveal lipid droplets using fluorescence (green) excites at 493 and emits at 503 (FITC channel 490/525).

### Western blotting

Western blotting was performed to examine changes in PLIN2 expression in response to oleic acid. After 24 hrs treatment, cells were washed with cold PBS, samples were lysed in ice-cold RIPA lysis buffer containing 25 mM Tris- HCl pH 7.6, 150 mM NaCl, 1% NP40, 1% sodium deoxycholate, 0.1% SDS (Thermo Scientific) supplemented with 1% protease inhibitor. Total protein (20 μg) was resolved on a 4–12% Bis-Tris polyacrylamide gels, and transferred to a nitrocellulose membrane (Amersham), blocked with 5% nonfat milk in Tris-buffered saline containing 0.2% Tween 20 for 1 hrs. Blots were then incubated with PLIN2 (1:600, #NB110-40877 Novus)^46^ primary antibody overnight at 4 °C. The membranes were then stripped and incubated with GAPDH (1:2000, #MAB374 Millipore)^47^. Antibodies were detected by secondary antibody incubation (HRP-goat anti rabbit and anti mouse, Santa Cruz Technology) for 1 hrs at room temperature. Chemiluminescence (Pierce ECL, Thermo scientific) was quantified on scanned films using Image J software.

### RNA and real-time quantitative PCR

U-138 GBM cells grown in 60 mm dish were rinsed with ice-cold PBS before RNA extraction using Trizol method (Invitrogen). RNA concentration was quantified spectrophotometrically. Following the manufacturer’s conditions, 2 µg of total RNA was reverse transcribed by SuperScriptII reverse transcriptase (Invitrogen) with oligo (dT). Quantitative gene expression was measured from 1 µl of cDNA. Real-time PCR was performed using the ABSYBR green PCR Master Mix, according to the manufacturer’s guidelines. Data were normalized to 18 S expression levels and analyzed using the Delta CT method. The list and sequences of primers is provided in Table 1.

**Table 1:** Primers used for real-time quantitative PCR.

Gene	Forward primer	Reverse primer
CPT1-A	GTTCTCTTGCCCTGAGACGG	TTTCCAGCCCAGCACATGAA
CPT1-c	CCTGGTGGGCGTCCAATTAT	GCGAGCTGCCATCATGTAAT
FASN	CAGAGCAGCCATGGAGGAG	TCCTGCAAGTTCTCCGACTC
GPAT2	CTCCTTCCCTTGAGCAGTCC	TCACACCAGCTTCCAACTCC
DGAT2	GCCCCGTTGTGAGGTGATAA	GAAACAGCCTAGACCCCAGC
DGAT1	AAGTGCTGTCCAGTGACCTC	TCCACACAGCTCTGGCACTC
SREBP	GATGGACGAGCCACCCTTC	GCCGACTTCACCTTCGATGT
PLIN2	GCCATTGTGTTGCCTCTGTTA	ACGCCTTTCAGATCACACCA
PLIN3	GGGCCCCGTCTCTATAATGC	AGTGGACAGCACAGAAGAGC
18S	CGTCGTCCTCCTCGCTTG	TAGGTAGAGCGCGGCGA

DGAT1 and 2, diacylglycerol acyltransferase 1 and 2; GPAT2, glycerol-3-phosphate acyltransferase 2. FASN, fatty acid synthase and SREBP, sterol regulatory element-binding protein-1c, CPT1, Carnitine palmitoyl transferase, PLIN, Perilipin.

### LCFA oxidation in U138-MG GBM cells

The measurement of LCFA oxidation in U138 cells was performed as previously described^35^. Briefly, 0.7 × 10^6^cells were seeded in T25 flasks. After 24 hrs, media was removed and cells were starved for 2 hrs in serum free EMEM with 5 mM glucose, followed by a 30 min pre-incubation in the same media supplemented with 0.13% BSA. Cells were then incubated for 4 hrs in presence of 5 mM glucose, 0.1 mM oleic acid pre-complexed to 0.13% BSA [1–14 C]-Oleic acid (0.1 μCi/ml). The flasks were sealed at the beginning of the incubation with a stopper containing a filter (Whatman GF/B paper) pre-soaked in 5% KOH. The reaction was stopped by the injection of 0.2 ml 40% perchloric acid into each flask via a needle through the cap to acidify the medium and liberate the CO_2_. After overnight isotopic equilibration at room temperature, filters were removed and the trapped ^14^CO_2_ and ^14^C-Acid Soluble Products were counted to estimate total oleic acid oxidation using the liquid scintillation analyzer Tri-Carb 4810 TR. The results were normalized by cell protein content.

### Glucose utilization in U138-MG GBM cells

0.7 × 10^6^ cells were seeded in T25 flasks. After 24 hrs, media was removed and cells were starved in 1 mM glucose Krebs-Ringer bicarbonate HEPES (KRBH) buffer of the following composition: 135 mM NaCl, 3.6 mM KCl, 5 mM NaHCO_3_, 0.5 mM NaH_2_PO_4_, 0.5 mM MgCl_2_, 1.5 mM CaCl_2_, and 10 mM HEPES, pH 7.4 for 2 h, and pre-incubated in KRBH 1 mM glucose containing 0.13% BSA during 30 min. Cells were then incubated in KRBH 5 mM glucose, in presence or absence of 0.1 mM oleic acid pre-complexed to 0.13% BSA, D-[U-3H]-glucose (0.2 μCi/ml). At the end of the incubation, the medium was collected and acidified with concentrated HCl (10% of volume). Samples were placed in scintillation vials containing cold water and incubated for 24 hrs at 50 °C under constant agitation. After equilibration, ^3^H2O was counted to calculate glucose utilization. The results were normalized by cell protein content.

### Cell proliferation essay

1.4 × 10^5^ cells were seeded on coverslips in the 12 well plate and incubated in complete media (5 mM Glucose) supplemented with 10% FBS. After 24 hrs, the media is removed and cells are incubated in serum deprived media (5 mM glucose) for 24 hrs in presence of 0.1 mM oleic acid precomplexed to 0.13%BSA, and different concentrations of dietheyl-p-nitrophenylphosphate, JZL 148, or etomoxir. EdU was added 3 hrs before the end of the incubation; cells were then fixed and stained for EdU (red) and DAPI (blue). Cells were observed with Nikon eclipse fluorescent microscope and counted using NIH Image J software.

### Cell migration assay

Cell migration activity was evaluated by the Radius 24-well assay kit (Cell Biolabs, San Diego, CA, USA), according to the manufacturer’s instructions. Briefly, 1.7 × 10^5^ cells were seeded per well in the 24 well plate with a circular biocompatible gel in the center of each well and incubated to allow their attachment. After 12 hrs, the biocompatible gels were removed with removal solutions, and the cells were incubated for an additional 24 hrs in presence or absence of oleic acid (0.1 mM). Images were taken in bright field illumination.

### Thin layer chromatography

Oleic acid accumulation into neutral lipids was measured using thin layer chromatography (TLC). Cells were incubated during 24 h in the presence of 0.1 oleic acid pre-complexed to 0.5% BSA, at the end of the incubation cells were collected, washed with cold PBS, and pellet suspended in PBS 1% propanol. Same amount of proteins was taken and total lipids were extracted using the Folch method. Briefly, samples were loaded into pre-chilled glass tubes containing 1 ml of Folch reagent (2 chloroform: 1 methanol). 6 N HCl was added, and samples were vigorously vortexed for 1 min, incubated on ice for 10 min and centrifuged at 3000 rpm for 15 min at 4 °C. After centrifugation, the lower phase (organic) was transferred into pre-chilled glass tubes and dried under N2. Each sample was suspended in 50 μl of chloroform and loaded on the TLC plates (Whatman). The samples were delivered by small drops, Total lipids were separated using a solvent for neutral lipids (petroleum ether/ether/acetic acid; 80:20:1.5), after separation the plate was dried under hood for 15 min then placed in iodine chamber 2–3 min. the plate was completely sprayed with 8% phosphoric acid 3% cupric sulfate solution and incubated at 140° for 10 min.

### Mass spectrometry

Cells (1.0 × 10^6^) were seeded in T75 cells, in complete media (5 mM Glucose) supplemented with 10% FBS, After 24 hrs, the media was removed and cells were incubated in serum deprived media (5 mM glucose) in presence or absence of 0.1 mM oleic acid precomplexed to 0.13% BSA. After 24 hrs, cells were harvested in water, centrifuged at 12,000 rpm for 10 min and stored at −80 for mass spectrometry. Lipids composition in cells in response to oleic acid treatment was measured using TripleToF 5600 spectrometer. Non-esterified fatty acids, was measured using gas chromatoghraphy 59/75 mass spectrometry. Small volume is kept for protein quantification to normalize results. Figures are prepared with Python 3.6.4 software.

### MAG extraction

Monoacylglycerol (MAG) from astrocytic cell samples were performed using a modified Bligh & Dyer^48^ method. Briefly, cell pellets were lysed and homogenized in water followed by the addition of methanol and chloroform. After the clear phase separation, organic layers containing lipids including MAGs were collected and dried in a nitrogen evaporator (Organomation, Berlin, MA, USA) and stored at −80 °C. Dried extracts were reconstituted with methanol containing MAG internal standard (MAG 17:1) at a concentration of 100 ng/mL prior to analysis.

### LC-ESI-MS/MS analysis of MAGs

Targeted analysis was performed to quantify the MAGs levels in astrocytes samples. Briefly, chromatographic separations of MAGs present in the cell samples were performed on a C18 reverse-phase column (2.6 µm, 50 × 2.1 mm) with an ULTRA HPLC In-Line Filter (0.5 µm Depth Filter x 0.004 in ID) (Phenomenex, Torrance, CA, USA) using a Shimadzu ultra fast liquid chromatography (UFLC) system (Shimadzu, Nakagyo-ku, Kyoto, Japan) coupled to a hybrid triple quadrupole LIT (linear ion trap) mass spectrometer 4000 QTRAP system equipped with Turbo Ion Spray (SCIEX, Foster City, CA, USA). Electrospray Ionization (ESI, + ve) was used to ionize these lipid species and individual MAG species was detected by multiple reaction monitoring (MRM). Mass spec conditions were as follows: the ion spray voltage (V) was 4,500 at ion source temperature of 550 °C with a collision gas of 9 psi, and curtain gas of 20 psi. The declustering potential was 50 V, the entrance potential 10 V, the collision energy 15 eV, and the collision cell exit potential was 15 V. The mobile phase consisted of A: 10 mM ammonium formate in methanol: water (37:43 v/v) and B: 10 mM ammonium formate in methanol. The following gradient was used: 0.0–2.0 minutes at 10–100% B, hold for 5 minutes (2–7.5 minutes) at 100% B, then 7.5–9 minutes 10% B, 9.0–10 minutes 100% B with a flow rate was 0.45 ml/min. Each MAG was quantified based on the spiked internal standard (MAG 17:1) concentration. Instrument control and data acquisition were performed by using Analyst (version 1.4.2, SCIEX Inc. Thornhill, Ontario, Canada) and data analysis were completed using MultiQuant software (version 2.0, SCIEX, Thornhill, ON, Canada).

## Supplementary information

Supplementary Dataset1
