# Supplementary material for: Lipid accumulation and oxidation in glioblastoma multiforme
Source: Sci Rep. 2019 Dec 20;9:19593. doi: 10.1038/s41598-019-55985-z (PMC6925201; doi:10.1038/s41598-019-55985-z)
Supplement: Supplementary file 1 — Supplementary Dataset1 [file 41598_2019_55985_MOESM1_ESM.pdf]

Lipid accumulation and oxidation in glioblastoma multiforme

Bouchra Taïb[1], Amine M. Aboussalah[2], Mohammed Moniruzzaman[3], Suming Chen[3], Norman J. Haughey[3], Sangwon F. Kim[1,4], and Rexford S. Ahima[1]

1-Department of Medicine, Division of Endocrinology, Diabetes and Metabolism, Johns Hopkins University, Baltimore, Maryland, U.S.A

2- Department of Mechanical & Industrial Engineering, University of Toronto, Toronto, Canada

3- Department of Neurology, Johns Hopkins University, Baltimore, Maryland, U.S.A

4- Department of Neuroscience, Johns Hopkins University, Baltimore, Maryland, USA

Supplementary figure S1

PLIN2

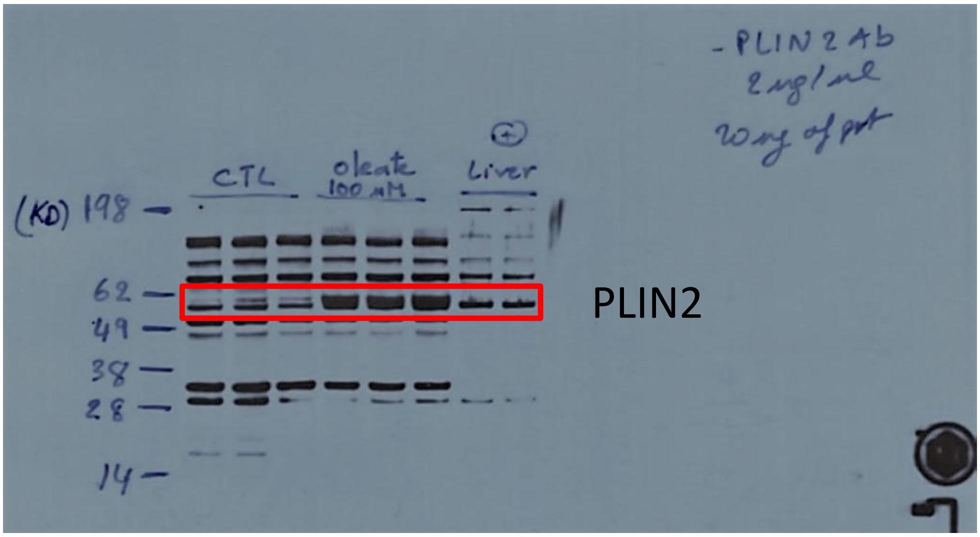

GAPDH

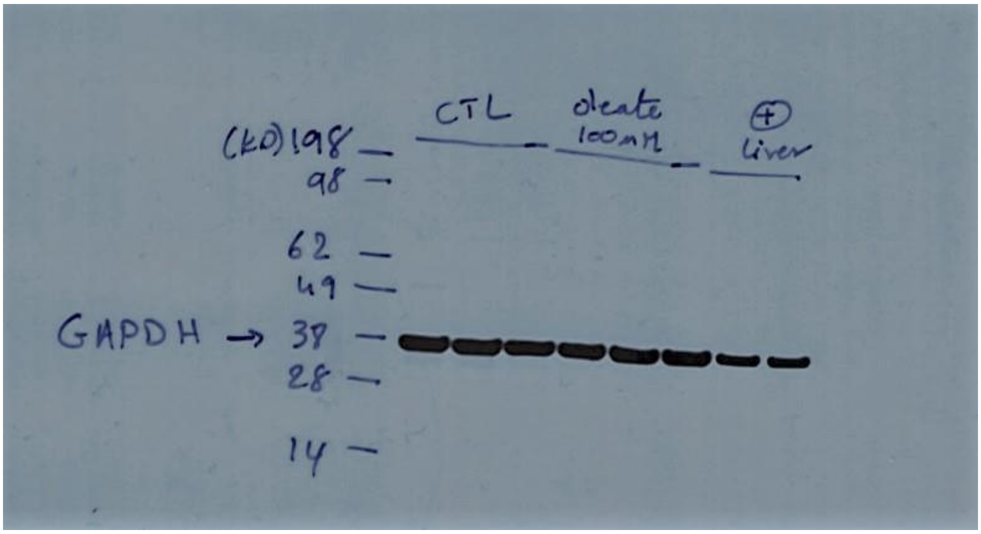

Supplementary Figure S1: Full-length blot images for cropped western blot presented in figure 1-B Perilipin-2 Antibody was purchased from (Novus, NB110-40877)

Supplementary figure S2

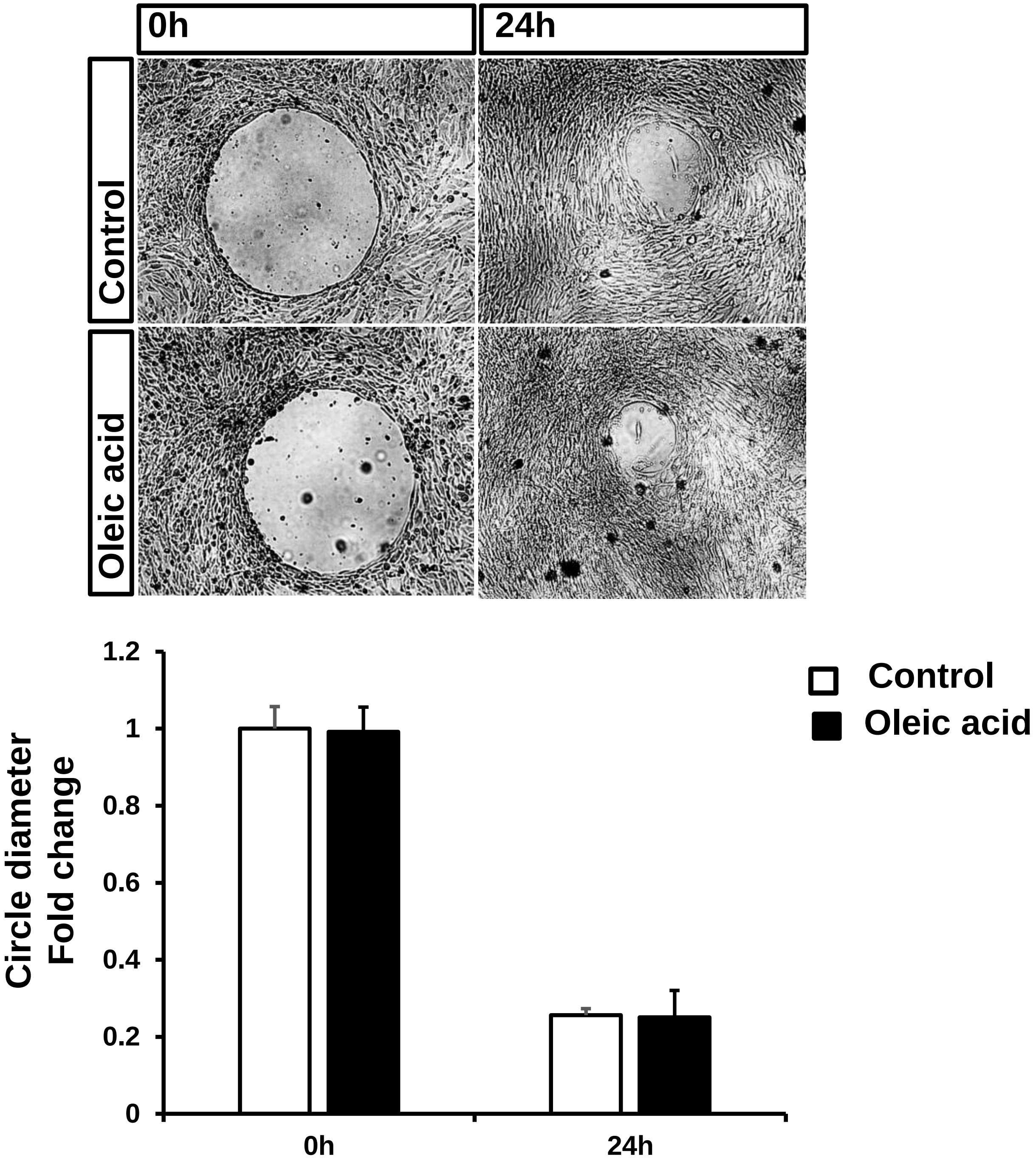

Supplementary figure S2: : Effect of oleic acid on U138 GBM cells migration

Migration was evaluated using Radius cell migration assay performed following the manufacturer's protocol. Images were captured at the same magnification at the beginning of the migration and after 24 h. Values were means ± SE. N=3 independent experiments.

Supplementary figure S3

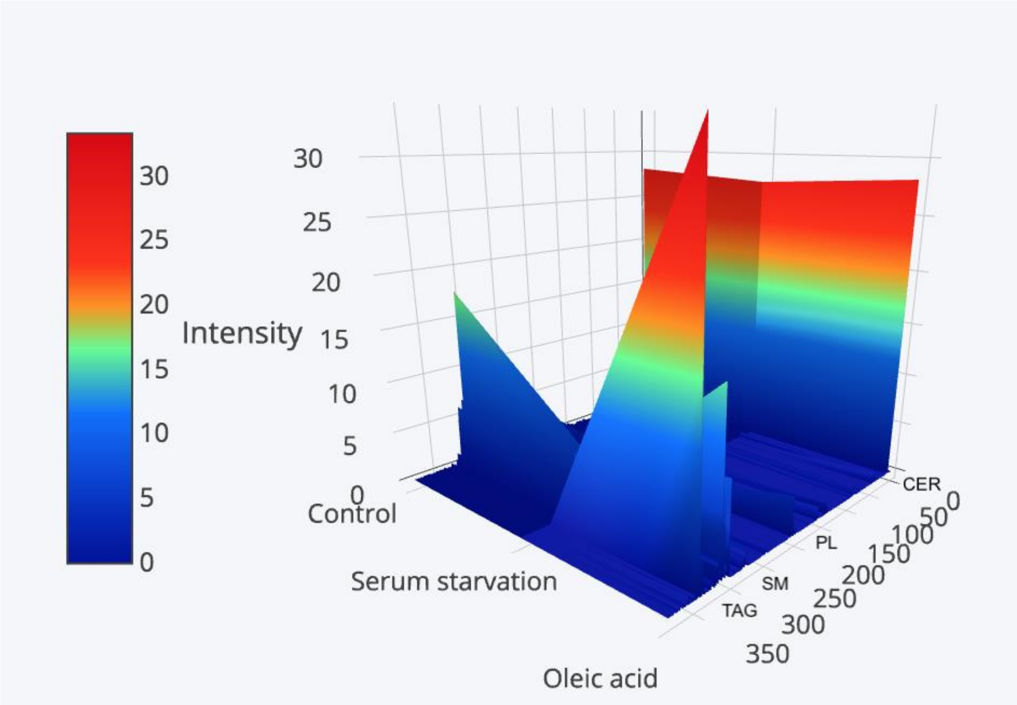

Supplementary Figure S3: 3D lipid mapping in U138 GBM cells
3D mapping summarizing the lipid species distribution in U138 GBM cells in response to 24hours serum starvation+/- oleic acid treatment, N=3. Results are presented in (ug/mg). TAG: Triglyceride, PL: Phospholipid, CER: Ceramides,SM: Sphingomyelin.

Supplementary Table S1

Lipids		
**Triacylglycerols**	**Control**	**Oleic acid**
TAG 54:1 (-FA 22:1)	0.016 ± 0.005	0.014 ± 0.002
TAG 54:3 (-FA 12:1)	0.008 ± 0.002	0.01 ± 0.001
TAG 54:3 (-FA 14:0)	0.025 ± 0.007	0.026 ± 0.003
TAG 54:3 (-FA 16:0)	0.005 ± 0.003	0.015 ± 0.003
TAG 54:3 (-FA 16:2)	0.019 ± 0.002	0.018 ± 0.002
TAG 54:4 (-FA 12:1)	0.053 ± 0.012	0.05 ± 0.001
TAG 54:4 (-FA 16:0)	0.011 ± 0.006	0.025 ± 0.005
TAG 54:4 (-FA 16:1)	0.002 ± 0.002	0.011 ± 0.001
TAG 54:4 (-FA 18:1)	0.021 ± 0.002	0.489 ± 0.072
TAG 54:4 (-FA 20:3)	0.01 ± 0.005	0.03 ± 0.007
TAG 54:4 (-FA 20:4)	0.013 ± 0.001	0.017 ± 0.002
TAG 54:5 (-FA 18:1)	0.015 ± 0.003	0.055 ± 0.008
TAG 54:5 (-FA 18:2)	0.011 ± 0.003	0.028 ± 0.008
TAG 54:5 (-FA 20:4)	0.018 ± 0.002	0.067 ± 0.014
TAG 54:6 (-FA 16:1)	0.002 ± 0.001	0.018 ± 0.005
TAG 56:0 (-FA 16:0)	0.038 ± 0.009	0.043 ± 0.004
TAG 56:0 (-FA 18:0)	0.023 ± 0.006	0.019 ± 0.001
TAG 56:3 (-FA 18:2)	0.015 ± 0.003	0.013 ± 0.001
TAG 56:3 (-FA 22:1)	0.014 ± 0.002	0.015 ± 0.001
TAG 56:5 (-FA 16:0)	0.011 ± 0.002	0.032 ± 0.007
TAG 56:6 (-FA 20:3)	0.001 ± 0.001	0.011 ± 0.002
TAG 56:7 (-FA 20:4)	0.003 ± 0.002	0.019 ± 0.004
TAG 58:2 (-FA 24:0)	0.015 ± 0.004	0.016 ± 0.004
TAG 58:3 (-FA 16:0)	0.023 ± 0.007	0.028 ± 0.002
TAG 58:8 (-FA 20:3)	0.006 ± 0.004	0.006 ± 0.001
TAG 62:5 (-FA 20:2)	0.016 ± 0.005	0.013 ± 0.001
TAG 66:3 (-FA 24:0)	0.006 ± 0.00	0.01 ± 0.002
**Phosphatidylcholines**	**Control**	**Oleic acid**
PC 32:5	0.001 ± 0	0.001 ± 0
PC 34:4	0.007 ± 0.002	0.007 ± 0.001
PC 44:0	0.002 ± 0	0.009 ± 0.002
PC 44:3	0.001 ± 0	0.001 ± 0

Supplementary Table 1: Additional lipid species in U138 GBM cells
The table shows additional TAG and Phosphatidylcholine species without changes in their expression in response to oleic acid treatment. TAG: Triglycerides, PC: Phosphatidylcholine. N=3.

Supplementary figure S4

A **U138 GBM cells**

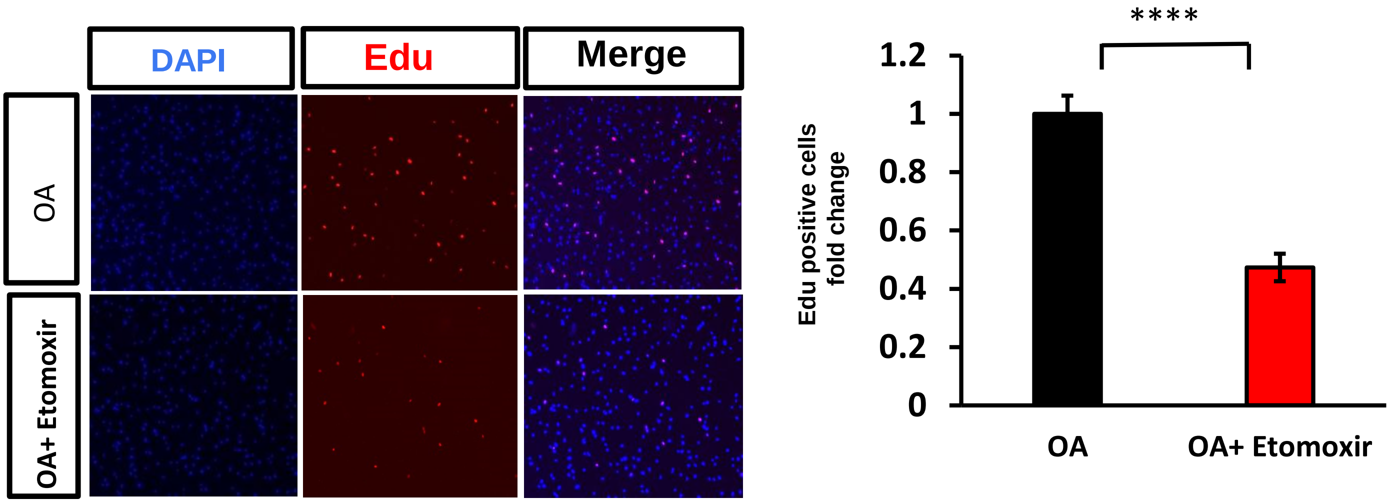

B **CRL8621 astrocytic cells**

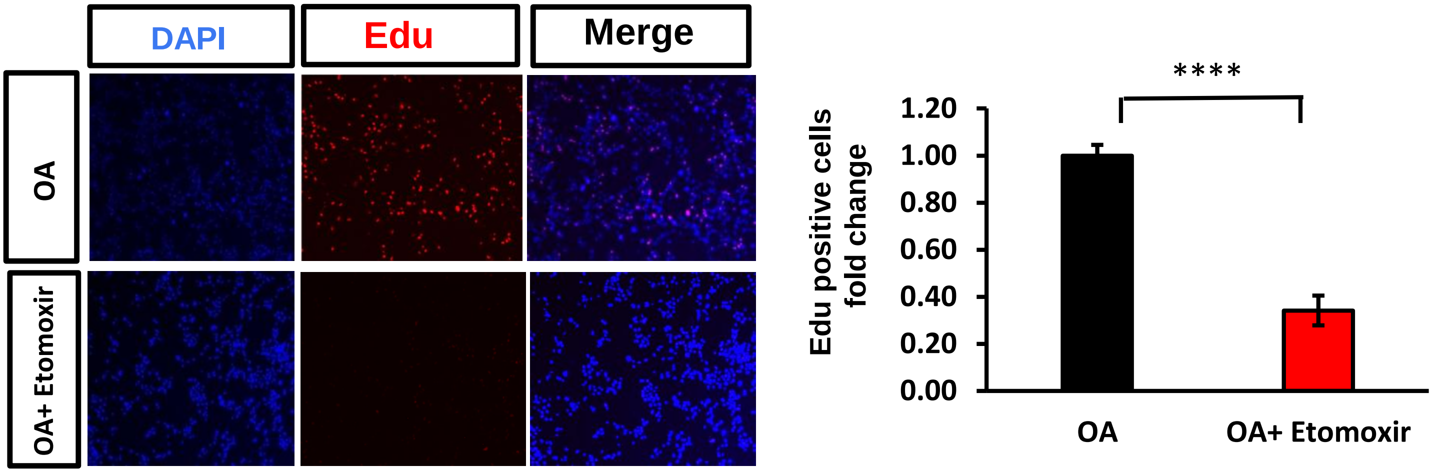

Supplementary figure S4: Effect of Etomoxir on U138 GBM and CRL8621 astrocytic cells proliferation

A- EDU proliferation test was performed in presence of oleic acid (OA, 100 µM) in response to Etomoxir (200 µM) in U138 GBM cells. **B**- EDU proliferation test was performed in presence of oleic acid (0.1mM) in response to Etomoxir (200 µM). Proliferating cells were detected via EDU (red). DAPI stained nuclei in blue. Merged view of EdU (red) and DAPI (blue). N=3 independent experiments. Statistical analyses were performed with unpaired Student's t-test **** $p < 0.0001$ versus control.

Supplementary figure S5

A

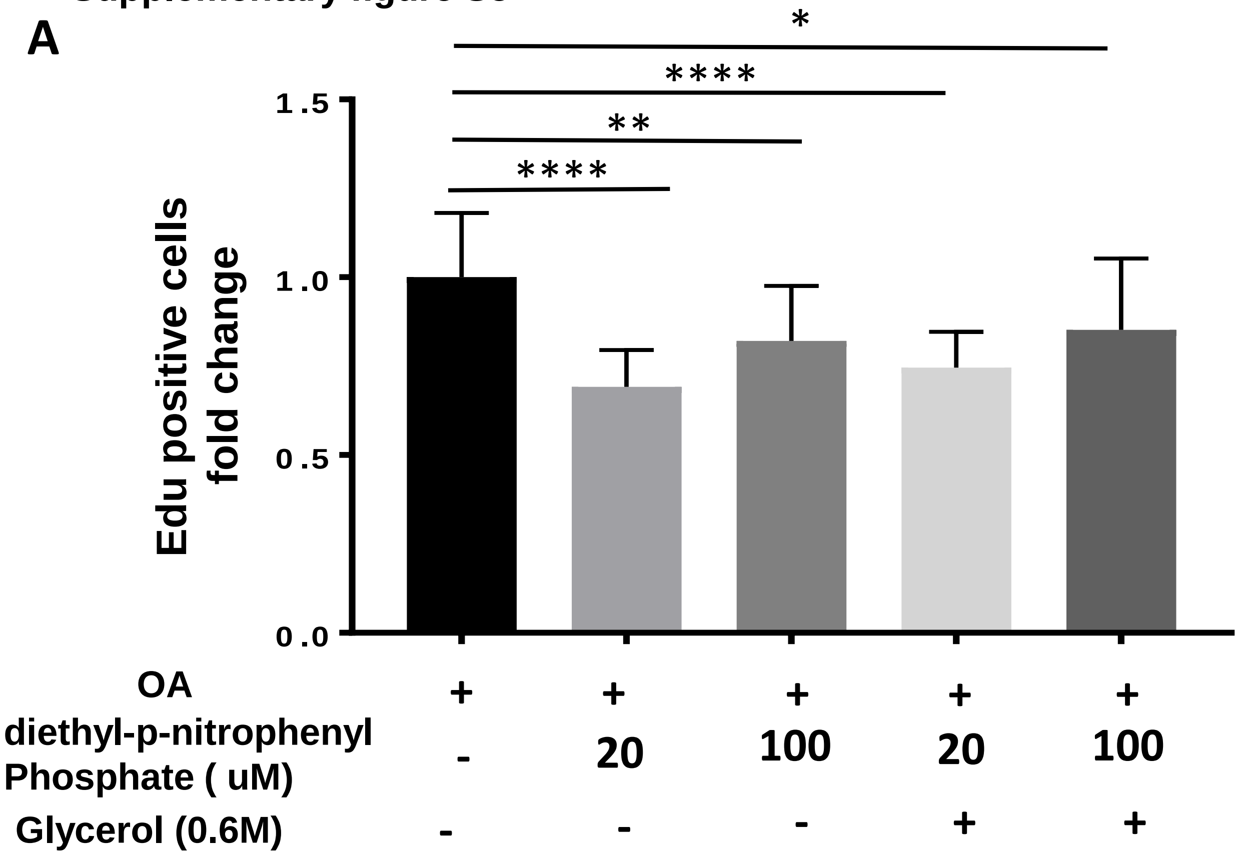

B

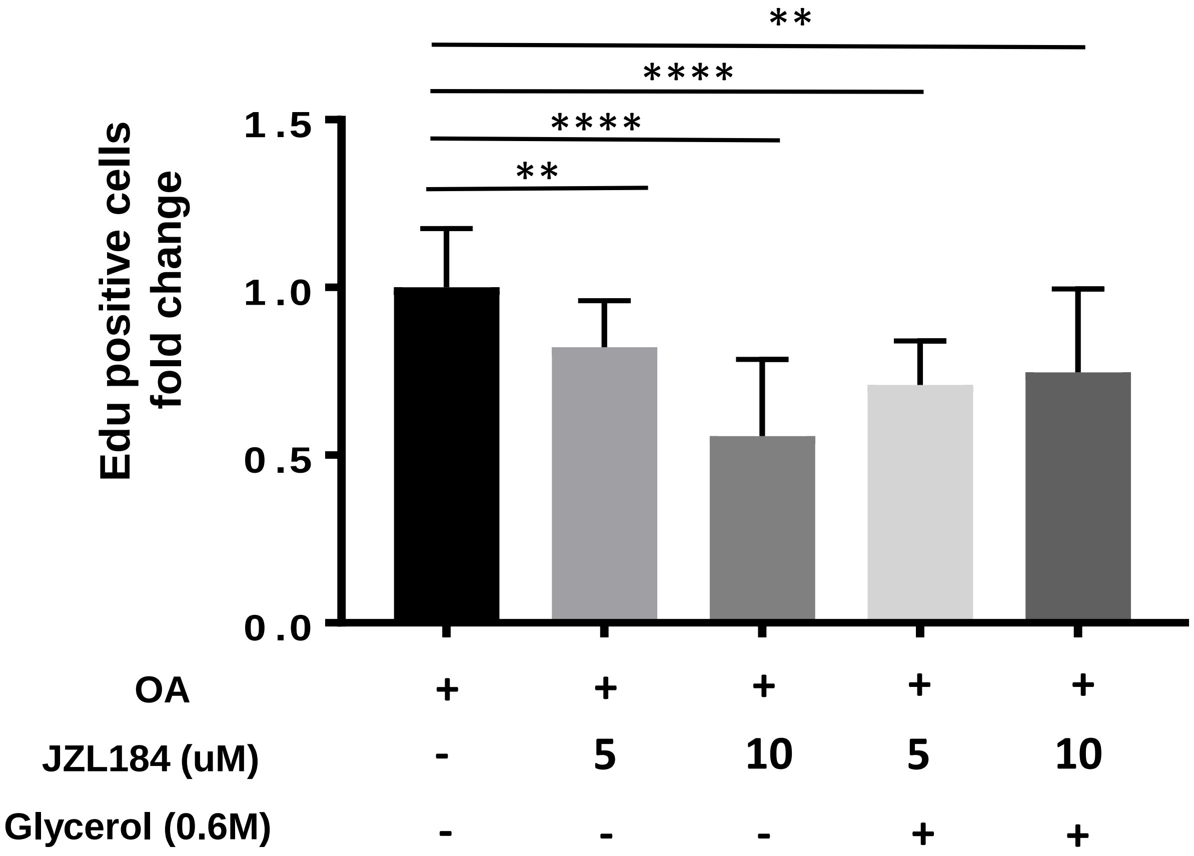

Supplementary figure S5: Glycerol does not show a difference in diethyl-p-nitrophenylphosphate and JZL 184 effects on U138 GBM proliferation

A- EDU proliferation test was performed in presence of oleic acid (OA, 100 µM) in response to different concentrations of the general lipase inhibitor diethyl-p-nitrophenylphosphate (20 µM, 100 µM) +/- glycerol (0.6M). **B-** EDU proliferation test was performed in presence of oleic acid (0.1mM) in response to different concentrations of JZL (5µM, 10µM) +/- glycerol (0.6M). Quantification was performed using Image J software. Proliferating cells were detected via EDU. N=3 independent experiments. Statistical analyses were performed with unpaired Student's t-test *, **, and **** $p < 0.05$, 0.01 and 0.0001 versus control.
